# Multidimensional analysis of social discourse on Twitter (X) around Venezuelan migration in Colombia

**DOI:** 10.1371/journal.pone.0360050

**Published:** 2026-10-14

**Authors:** Joseph Martínez, Melissa Miller-Felton, Jose J. Padilla, Erika Frydenlund, Katherine Palacio

**Affiliations:** 1 Virginia Modeling, Analysis, and Simulation Center, Old Dominion University, Suffolk, Virginia, United States of America; 2 Department of Industrial Engineering, Universidad del Norte, Barranquilla, Colombia; University of Exeter, UNITED KINGDOM OF GREAT BRITAIN AND NORTHERN IRELAND

## Abstract

Colombia has become the principal host of Venezuelan migrants in Latin America, prompting a complex mix of solidarity, anxiety, and discrimination. This study offers a multidimensional analysis of Twitter (now X) discourse on Venezuelan migration in Colombia between 2014 and 2022, using natural language processing, geolocation inference, sentiment analysis, and manual annotation of derogatory terms. Drawing from 7.4 million posts, we examine how online discourse evolves across time, space, and language. While negative sentiment is pronounced in original tweets during periods of political and social unrest, our findings highlight that users tend to amplify neutral and positive messages—over 67% of retweets fall into these categories—suggesting an informal counterbalance to polarized discourse. Additionally, the analysis of the derogatory terms *veneco* and *veneca* reveals significant gendered patterns: the feminine form veneca appears more frequently in negative contexts, pointing to the gendered dimensions of xenophobic language. These results underscore the dual function of social media as a site of both stigmatization and resistance, offering insights into the emotional and cultural dimensions of migration discourse in Colombia’s digital public sphere.

## 1. Introduction

Social media, like X (formerly Twitter), provides a window for observing how attitudes towards migrants evolve across time, location, and socio-political context. Prior research suggests that social media platforms can also function as “echo chambers,” where polarized narratives about migrants circulate and reinforce each other [1,2]. In these online spaces, migrants may alternately be portrayed with empathy or suspicion and accompanying stigmatizing stereotypes.

The outflow of refugees and migrants from Venezuela is one of the largest human displacements in the world, where Colombia hosts approximately 2.81 million (36%), making it the principal host country in the region [3]. Migrants have been portrayed as negatively in media as influencing local job markets and unemployment, community insecurity, and pressure on public services [4,5]. Studies suggest that Venezuelan migration has been instrumentalized in political and media discourse, particularly during electoral debates, where it has often been framed as a social or security issue [6]. As Bulley [7] observes, although the Colombian government adopted relatively welcoming measures toward Venezuelan migrants, public reactions have been mixed.

Online social media discourse around the Venezuelan migration situation also reveals the intersection of xenophobia with gendered narratives. The literature on hate speech in digital spaces emphasizes that these expressions are not random; they often align with broader nativist discourses and can be exacerbated during times of social stress or political rhetoric that scapegoats migrants [8–10]. Research on migration discourse in social media further highlights the role of values and emotions in shaping online posts and demonstrates how hate speech can proliferate in networked publics [1,2]. These studies also underscore how layered prejudices can surface in online discussions, with xenophobia often entwined with sexism or other biases [11].

In the Colombian context, this intersection of xenophobia and sexism [12,13] warrants particular attention. In online discussions, particularly on social media, the derogatory slang terms *veneco* (masculine) and *veneca* (feminine) have evolved to become common references to Venezuelan migrants. While these terms can function as colloquial identifiers, they are also frequently associated with stigmatizing and gendered portrayals that reinforce social hierarchies and cultural boundaries between migrants and host community citizens. Understanding how such language operates requires attention to the cultural, linguistic, and socio-political context, for example, recognizing Colombian slang and local references within posts. Such expressions are not merely offensive; they can also reflect underlying xenophobic attitudes and contribute to the marginalization of the targeted group [14].

Several research initiatives have monitored these dynamics using large-scale social media data. For example, the *El Barómetro* (Formerly *El Barómetro de Xenophobia*) project documents recurring patterns of scapegoating and negative stereotyping in digital narratives about Venezuelan migrants in Colombia [15]. Other literature has shown that media, particularly in social media around migration topics, is impacted by contemporary events that reflect in negative sentiment spikes [16–18]. However, existing studies primarily focus on detecting the presence of such narratives rather than examining how they change structurally over time and across regions, or how platform dynamics (posting/reposting) shape which narratives are amplified.

By distinguishing between what users express and what becomes amplified through reposting behavior, this study provides new insight into how migration discourse about Venezuelans in Colombia is not only produced, but also reshaped in these digital environments. These findings extend existing work by identifying structural shifts in discourse, analyzing spatio-temporal variation in sentiment, and examining changes in thematic patterns over time.

## 2. Methodology

The proposed methodological framework includes four sequential stages, as outlined in Fig 1. These stages include (1) the extraction of posts; (2) filtering by geolocation and thematic relevance to Venezuelan migration; (3) the categorization of post sentiment through a semi-supervised model and manual annotation of derogatory terms’ sentiment; and (4) a multi-level analysis consisting of temporal and spatiotemporal sentiment analyses, topic dynamics in migration discourse, and a temporal analysis of the derogatory terms *veneco* and *veneca.* The methodological approach builds on [19] adapting in this study natural language processing and classification techniques applied to social media data to the analysis of migration discourse. The framework is adapted here to incorporate geolocation inference, spatio-temporal analysis, and targeted annotation of derogatory language about migration discourse in a host country. All datasets, preprocessing scripts, trained models, and analysis pipelines (including LDA topic modeling and changepoint detection) are publicly available in an open repository (10.17605/OSF.IO/J8P6S).

**Fig 1:**
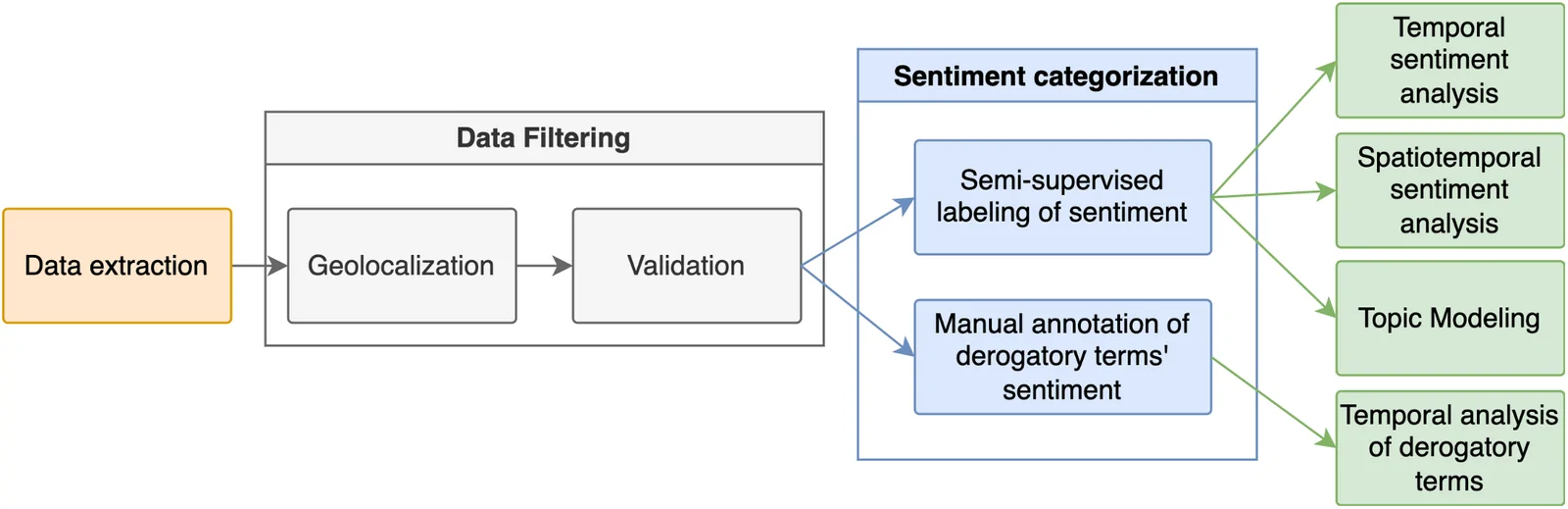
Methodology.

### 2.1. Data extraction

We collected data using the former Twitter Streaming API, and ran a search query containing keywords related to Venezuelan migration. The query retrieved content that refers to *migration* or *migrants*, in conjunction with terms identifying Venezuelan nationality (including *venezolano/a/os/as*, *Venezuela*, and common abbreviations such as *VZLA* or *VNZLA*), or that contains the derogatory terms of *veneco*, *venecos*, *veneca*, or *venecas*, accounting for plurality and grammatical gender (o/os for masculine and a/as for feminine) in Spanish (henceforth, we refer to these as the *masculine term* or *the feminine term*). The search retrieved not only original posts but also reposts and mentions referencing the target keywords. The extraction took place on July 4, 2022, and yielded a dataset comprising 7.4 million posts spanning the period from January 2014 to June 2022.

X users are not demographically representative of the general population. Advertising data indicate that X had approximately 4.3 million users in Colombia in early 2022, corresponding to about 8.4% of the national population [20]. Research also suggests that users of the platform tend to be younger and more educated than the broader public [21], which may lead to the underrepresentation of other demographic groups. Consequently, the dataset should be interpreted as reflecting the discourse of an active online community rather than the views of the entire population.

This study used publicly available, anonymized social media data and did not involve interaction with individuals or access to identifiable personal information. All data were fully anonymized prior to access. According to institutional guidelines, this study does not constitute human subjects research and therefore did not require ethical approval. The requirement for informed consent was waived, as the study relied exclusively on publicly available and non-identifiable data.

In this context, it is useful to distinguish between reposts and quote posts. Reposts correspond to verbatim reshares of an original post without additional user-generated text, whereas quote posts include new commentary appended to the original content. Our analysis focuses on original posts and reposts. Quote posts were excluded because their interpretation is more complex: users may quote a post to endorse, critique, or contradict its content, making sentiment attribution ambiguous without additional contextual analysis linking the quoted and original messages.

### 2.2. Data filtering

#### 2.2.1. Geolocalization.

From the extracted dataset, posts were first filtered by geographic origin. Of the 7.4 million posts, only 2.35 million had active geolocation data as user-defined location fields, while the remaining 5.1 million had no data associated. User-provided locations fields (e.g., “Bogotá,” with no mention of “Colombia”) were standardized using Nominatim API [22]. Of the 2.35 million, the most frequent countries were Venezuela (47%), Colombia (28.4%, with 353,776 posts), the United States (16.9%), and Spain (14.2%). As we wanted to capture the whole conversation in Colombia, we trained a binary classification model to determine whether a post was from Colombia or not based on its text and used it to automatically label the remaining 5.1 million with no user-defined location.

For this, we fine-tuned two Spanish-language NLP models: spaCy’s es_core_news_sm [23] and BETO (Spanish Bert) [24], a BERT variant trained on Spanish corpora; using a training dataset of 270,000 posts (135,000 from Colombia and 135,000 from other countries), a sample taken from the posts geolocated with the Nominatim API [22]. Further details on the model architecture, pre-training, fine-tuning procedures, and parameters are provided in S1 Text (Supplementary Material). All models were trained using a 70/30 train-test split [25]. The spaCy model achieved the highest classification accuracy at 0.79 and was therefore selected to infer geolocation for the remaining posts. The training results are presented in Table 1. Ultimately, 2 million posts were identified as originating from Colombia.

**Table 1:** Performance of the text geolocation models.

Architecture	Accuracy	Precision	Recall	F1
es_core_news_sm	0.79	0.795	0.794	0.794
BETO	0.50	0.250	0.500	0.333

Beyond overall accuracy, the selected geolocation classifier achieved a macro-averaged precision of 0.795, recall of 0.794, and F1-score of 0.794 on the held-out test set (Colombia: precision 0.789, recall 0.804, F1 0.796; other: precision 0.800, recall 0.785, F1 0.792). Because this classifier was applied to the approximately 5.1 million posts lacking user-provided location data, even this level of performance implies a non-negligible number of misclassified posts at the scale of the full corpus; we discuss the implications of this margin of error, together with the possibility that the classifier partly captures topic-specific or politically inflected vocabulary correlated with (rather than diagnostic of) geographic origin, in the Limitations section.

#### 2.2.2. Validation.

To ensure that our analysis focused exclusively on posts relevant to the Venezuelan migration, we developed a classification model to distinguish relevant from irrelevant posts. We first extracted a subset of 4,684 posts from our dataset for manual labeling. Each post was independently reviewed by human annotators and assigned a binary label: relevant (discussing Venezuelan migration) or irrelevant (unrelated to the topic). This process resulted in 2,990 posts labeled as relevant and 1,693 as irrelevant.

The labeled subset was then used to train and evaluate the classification model, which was subsequently applied to automatically label the remaining posts from Colombia in the dataset (approximately 2 million posts).

To prepare the data for model training, we addressed duplication and class imbalance. To address class imbalance, we applied undersampling to create a balanced training set. We chose undersampling over oversampling approaches (e.g., duplication or synthetic minority oversampling) because, for short social media posts, oversampling techniques risk amplifying idiosyncratic phrasing from a small number of minority-class posts or introducing synthetically generated text that does not reflect naturally occurring language; undersampling instead trains on only naturally occurring, human-written posts, at the cost of discarding some majority-class data. This process yielded 1,554 posts in each category, totaling 3,108 posts. The dataset was then partitioned using a 70/30 train-test split, with 2,170 posts allocated for training and 938 for evaluation.

Three models were fine-tuned for this task: spaCy’s es_core_news_sm, the BETO transformer model, and bert-base-multilingual-uncased-sentiment [26]. A detailed description of these models, including architecture and training configuration, is provided in S1 Text. Performance was assessed using classification accuracy on the held-out test set and are presented in Table 2. Among the models evaluated, spaCy’s es_core_news_sm exhibited the highest accuracy (0.90), outperforming both BETO (0.81) and the multilingual BERT variant (0.84). Due to its superior performance, spaCy’s model was selected to automatically label the remaining dataset, ensuring a consistent and high-quality identification of migration-relevant posts across the corpus.

**Table 2:** Performance of the Validation models.

Architecture	Accuracy	Precision	Recall	F1
es_core_news_sm	0.90	0.902	0.900	0.900
BETO	0.81	0.816	0.816	0.816
BERT-base multilingual-uncased-sentiment	0.84	0.842	0.842	0.842

After filtering for relevance, the final dataset used in the analysis comprised 1,664,903 posts. Of these, 647,733 correspond to original posts, while the remaining 1,017,170 are reposts. Thus, original posts represent approximately 38.9% of the corpus, while reposts account for 61.1%. This distribution indicates that reshared content makes up the majority of the observed activity in the corpus. These proportions describe the distribution of activity in the corpus and should not be interpreted as the proportion of unique users associated with each type of post. Accordingly, analyses based on original posts reflect a smaller subset of the conversation, whereas analyses including reposts capture broader patterns of content circulation and amplification. Because reposts are not independent observations and may be driven by a comparatively small number of widely shared original posts, all temporal analyses in this study (see “Temporal sentiment analysis” below) are reported separately for the full corpus and for original posts only (Figs 3 and 4); the structural breaks identified by changepoint detection are consistent across both conditions.

### 2.3. Sentiment and topic categorization

#### 2.3.1. Semi-supervised labeling of sentiment.

We employed a semi-supervised learning approach built upon a manual annotation to evaluate the sentiment expressed in posts about Venezuelan migration. A subset of 2,990 posts previously classified as relevant was annotated for sentiment by two independent coders. Each post was assigned a score on a 7-point Likert scale ranging from −3 (extremely negative) to +3 (extremely positive), with 0 representing a neutral sentiment. The labeling rubric was refined through iterative discussion among the research team to ensure conceptual clarity and coding consistency (See Fig 2).

**Fig 2:**
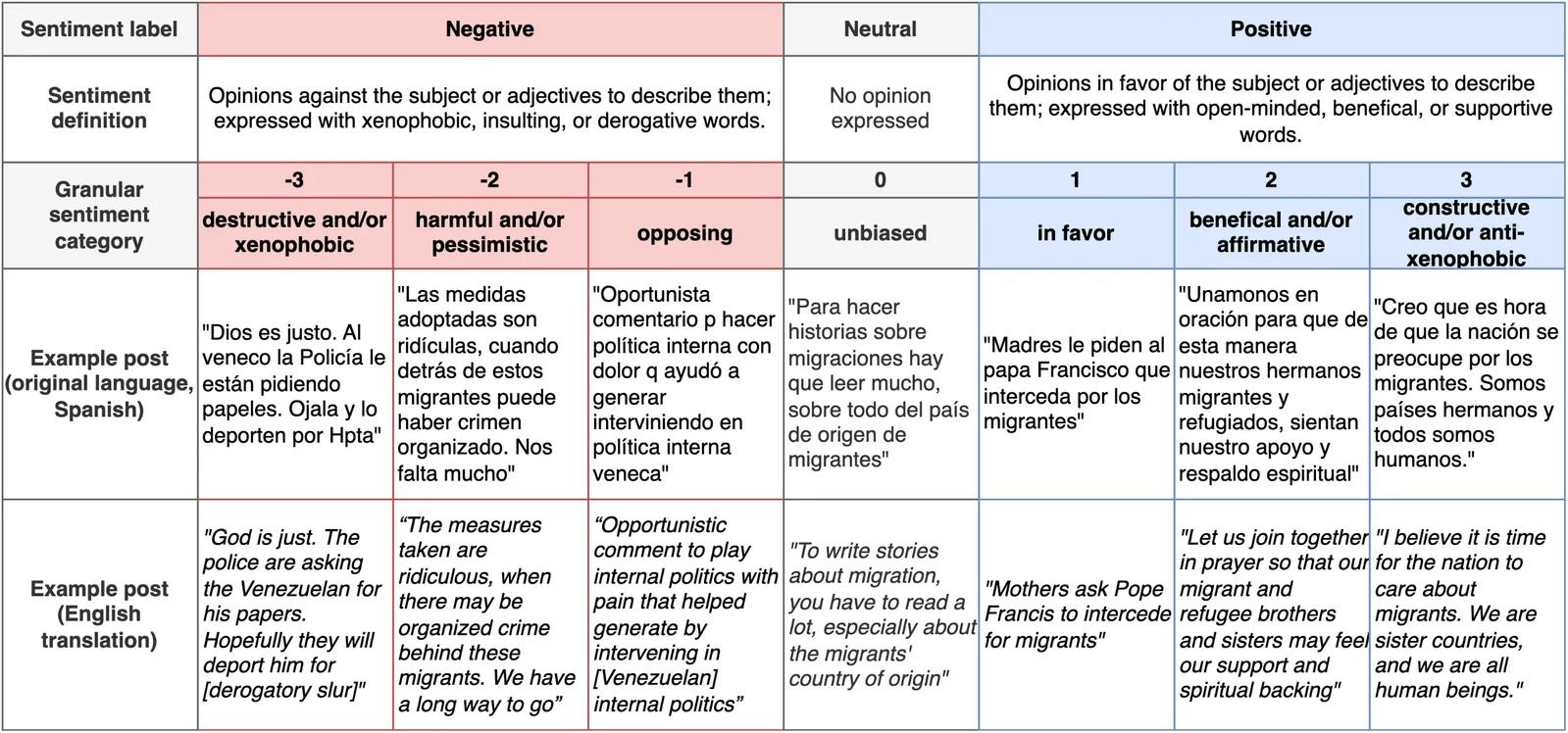
Criteria used for the sentiment scale labeling. Note: example posts are presented verbatim in Spanish; English translations preserve the original meaning while indicating offensive or derogatory terms using bracketed descriptors.

To reduce sparsity, scores were aggregated into three categories: negative (comprising −3 to −1), neutral (0), and positive (1–3). The intercoder agreement was calculated at 63.5% [27]. Disagreements were resolved through consensus. Because raw percentage agreement does not correct for agreement expected by chance, we also computed Cohen’s kappa on the original 7-point scale, which was κ = 0.56, indicating moderate agreement.

The final labeled corpus contained 1,920 posts and was split into training and testing subsets using a 70/30 ratio. We trained and evaluated three classification architectures on this task: es_core_news_sm, BETO, and bert-base-multilingual-uncased-sentiment (see S1 Text). As shown in Table 3, the Spanish BERT model achieved the highest accuracy (0.699) and was subsequently used for labeling the full set of migration-related posts. This model was selected for its superior performance and its pretraining on large-scale Spanish corpora, which allowed for more accurate interpretation of nuanced sentiment in the dataset.

**Table 3:** Performance of the sentiment classification models.

Architecture	Accuracy	Precision	Recall	F1
es_core_news_sm	0.552	0.556	0.552	0.553
BETO	0.699	0.707	0.700	0.700
bert-base-multilingual-uncased-sentiment	0.666	0.674	0.667	0.667

Beyond overall accuracy, the selected sentiment classifier’s per-class performance on the held-out test set was as follows: negative (precision 0.798, recall 0.656, F1 0.720), neutral (precision 0.655, recall 0.682, F1 0.668), and positive (precision 0.670, recall 0.760, F1 0.712), with a macro-averaged precision of 0.707, recall of 0.700, and F1-score of 0.700. This pattern indicates that the classifier is more conservative in predicting the negative class (higher precision, lower recall) than the neutral or positive classes, which should be kept in mind when interpreting the absolute levels of negative sentiment reported below; we discuss this, together with the broader distinction between sentiment and attitudes toward migrants, in the Discussion.

The Latent Dirichlet Allocation (LDA) [28] model identified eight topics in the corpus using the c_v_ coherence metric (c_v_ = 0.327) [29]. It was trained on a stratified sample of 200,000 original posts and subsequently applied to the full dataset to estimate monthly topic prevalence. The top keywords and descriptive labels associated with each topic are reported in Table S2-2 in the S2 Text supplementary material.

Candidate models were fit for topic counts ranging from 6 to 10 (Table S2-1 in S2 Text), and the eight-topic solution was selected because it achieved the highest coherence score among those tested (c_v_ = 0.327); solutions with fewer topics merged thematically distinct narratives (e.g., policy discussion and derogatory language), while solutions with more topics did not yield additional interpretable structure. This absolute coherence value is nonetheless modest, which is consistent with the well-documented difficulty of applying standard LDA to short, informally written social media posts that provide limited within-document word co-occurrence [30,31]; topic assignments should therefore be read as indicative thematic groupings rather than precise, mutually exclusive categories

To formally identify temporal shifts, we employed changepoint detection using the Binary Segmentation algorithm with Modified Bayesian Information Criterion (MBIC) penalty on log-transformed post counts (log(counts+1)) to stabilize variance [32].

#### 2.3.2. Manual annotation of derogatory terms’ sentiment.

In parallel with the general sentiment classification, we conducted a targeted manual annotation of posts containing the mentioned derogatory terms. This analysis aimed to capture these terms’ evolving connotations and affective load in digital conversations. Two independent coders reviewed and labeled each post containing either term, applying the same 7-point Likert scale used in the broader sentiment analysis (see

Fig 2). The criteria were adapted to focus on how the terms were used in reference to Venezuelan migrants, whether as neutral descriptors or as explicit slurs.

A total of 1,175 posts were annotated for this task: 870 containing the derogatory *masculine* term and 305 containing the derogatory *feminine* term. Following the same aggregation procedure as the general sentiment classification, the scale was collapsed into three categories: negative, neutral, and positive. Due to the limited number of posts involving these terms, this grouping allowed for greater analytical coherence while still preserving meaningful distinctions in sentiment. Intercoder reliability was 59.7% for the *masculine term* and 61.4% for the *feminine term.* Computing Cohen’s kappa on the same 7-point scale yielded κ = 0.48 for the masculine term and κ = 0.48 for the feminine term. While modest, these levels of agreement are consistent with existing research on manual sentiment labeling, particularly for emotionally charged or context-dependent expressions. The annotated data was subsequently used in descriptive temporal analyses of pejorative language, as presented in later sections of the paper.

The same changepoint detection procedure was also applied to identify structural shifts in the sentiment of posts containing the derogatory terms.

## 3. Results

This section presents four analyses of migration-related discourse on X: temporal patterns in post sentiment, spatial variation in sentiment across Colombian departments, the evolution of the derogatory terms, and the thematic structure of the conversation identified through topic modeling.

### 3.1. Temporal sentiment analysis

To examine how migration-related discourse evolves over time, we analyze differences between sentiment in original posts and reposts, and identify structural shifts using changepoint detection. We compare sentiment patterns in original posts and reposts to assess how amplification mechanisms shape them. We also examine temporal dynamics in sentiment and identify structural shifts in discourse using changepoint detection.

The inclusion of reposts substantially alters the sentiment distribution of the discourse (Fig 3a and b, respectively). While original posts skew more negative, reposted content is disproportionately neutral or positive: approximately 67% of reposts fall into these categories. This pattern suggests that users are more likely to share (repost) content that is not negative, systematically amplifying non-negative content in the overall online discourse about migration.

**Fig 3:**
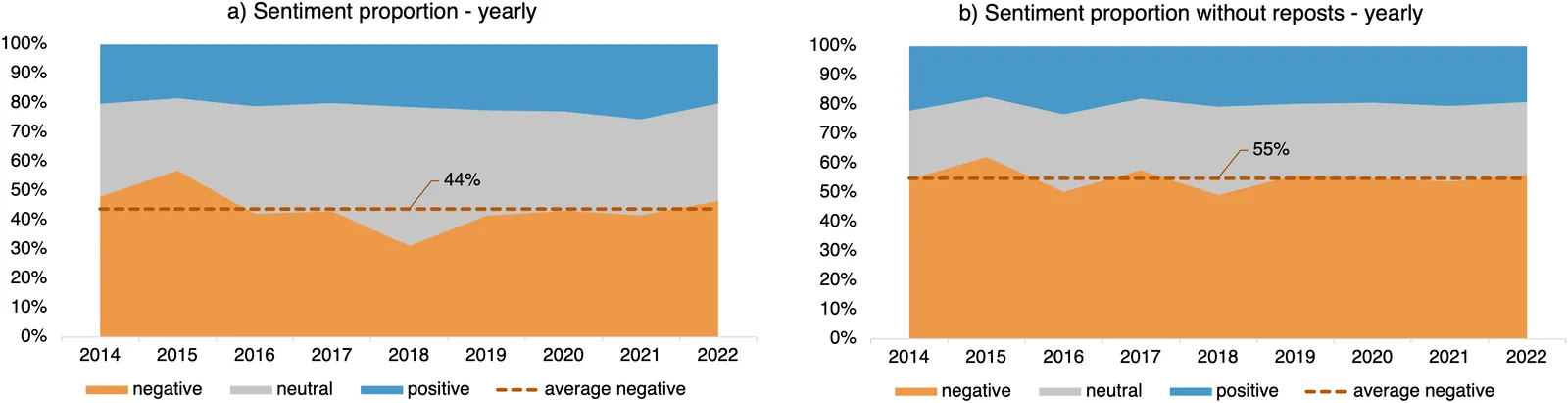
Yearly distribution of post sentiment categories from 2014 to 2022. (a) Distribution including original posts and reposts. (b) Distribution considering only original posts.

The monthly sentiment of the discourse varied considerably over time, with key inflection points aligning closely with sociopolitical developments (see Fig 4). Because the three sentiment categories are plotted on the same scale, their temporal variations can be directly compared without additional normalization. To formally identify these shifts, we employed changepoint detection and this revealed statistically significant structural breaks in sentiment patterns during 2017–2018, marking a fundamental transition from a lower-volume, more balanced discourse to a higher-volume regime in which negative sentiment becomes more prominent.

**Fig 4:**
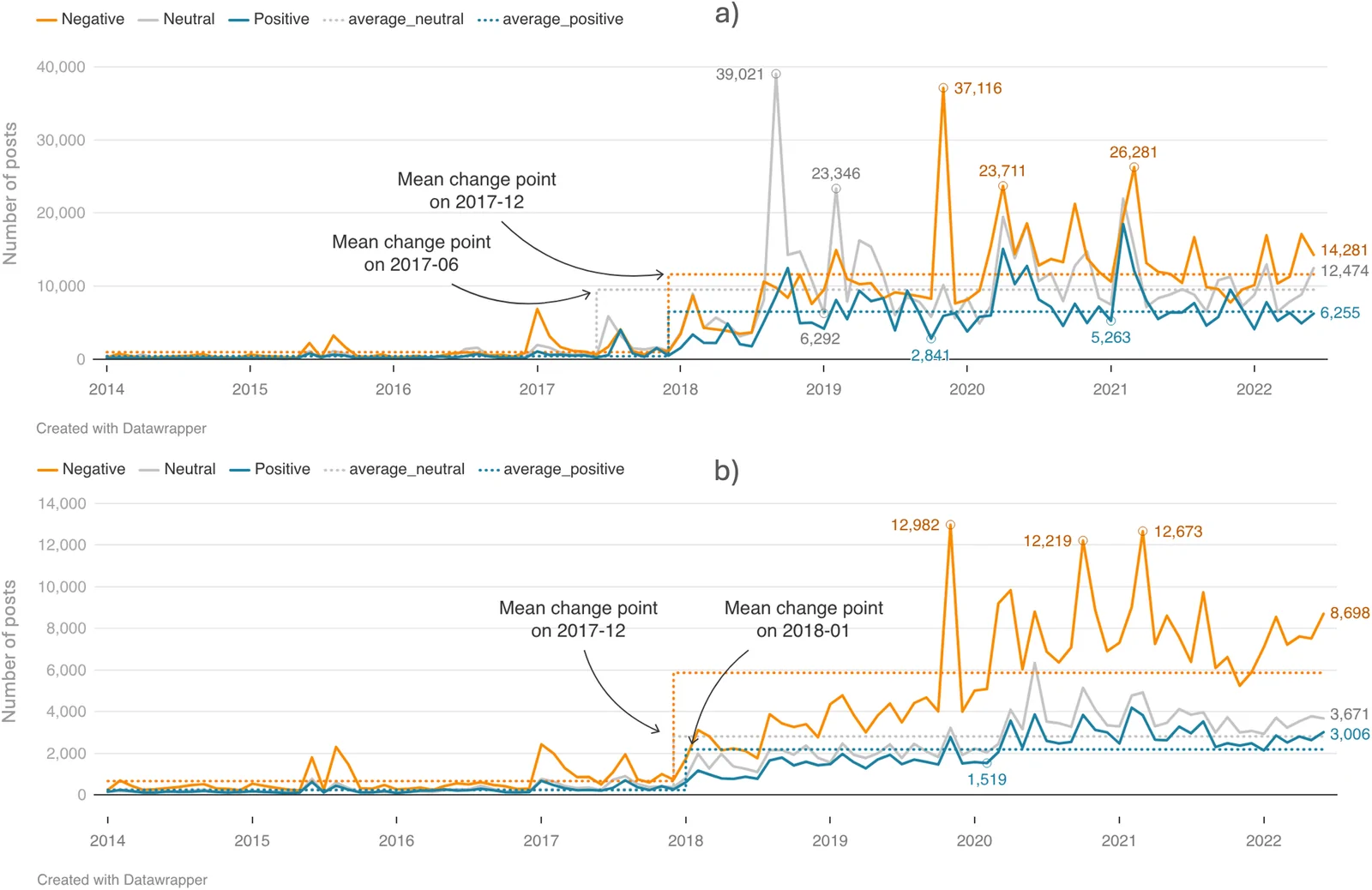
Monthly sentiment frequency (2014–2022). (a) Monthly frequency of post sentiment including original posts and reposts. (b) Monthly frequency of post sentiment considering only original posts.

Table 4 reports the complete results for both conditions. All detected breaks fall within the June 2017–January 2018 window and are highly significant (all p < 10^−30^), with post-break monthly volumes exceeding pre-break means by 797–1,843% depending on sentiment category and whether reposts are included. From 2014 to 2016, post volumes were relatively low, and sentiment was more evenly distributed. Neutral posts, reflecting factual reporting by news outlets and users alike, formed the plurality. However, 2017 and 2018 marked a clear shift: negative posts increased considerably, particularly in the period leading to the 2018 Colombian presidential election debates around migration [6]. Polarization increased, but while negativity rose, reposts amplified a more moderate sentiment.

**Table 4:** Changepoint detection results for monthly sentiment counts (2014–2022).

	Series	Changepoint	Pre-break mean	Post-break mean	Change	*p value*
Including reposts	Negative	Dec 2017	992	11,615	+971%	1.02 × 10^−31^
	Neutral	June 2017	490	9,525	+1,843%	5.09 × 10^−33^
	Positive	Dec 2017	429	6,513	+1,419%	1.46 × 10^−35^
Original posts only	Negative	Dec 2017	653	5,860	+797%	6.10 × 10^−32^
	Neutral	Dec 2017	270	2,800	+937%	1.49 × 10^−36^
	Positive	Jan 2018	221	2,175	+883%	8.87 × 10^−41^

To assess whether this national structural shift is an artifact of the text-based geolocation classifier rather than a genuine pattern, we repeated the changepoint procedure using only the subset of posts with a directly user-provided location, rather than an inferred one (n = 167,986 posts, 2014−01–2020−04; this is the same directly-geolocated subset used for the department-level analysis below), aggregated nationally by month. Applying the identical Binary Segmentation/MBIC procedure to this subset yielded structural breaks in the same months as the full-corpus results reported above (negative: December 2017; neutral: June 2017; positive: December 2017), indicating that the national-level structural shift is not an artifact of geolocation-inference error, at least within the window for which directly-geolocated data are available (S1 Fig). This comparison could not be extended to 2020–2022, because the directly-geolocated subset does not cover that period; see Limitations.

Negativity spiked in November 2019, around the time of the expulsion of 59 Venezuelans accused of inciting unrest. A similar negativity spike occurred in early 2020 during the onset of the COVID-19 pandemic. In contrast, a positivity increased in February 2021, shortly after the announcement of the *Estatuto Temporal de Protección para Migrantes Venezolanos* (ETPV), a policy granting undocumented migrants access to legal status and public services.

Original posts reveal a consistently higher proportion of negative sentiment across the entire study period compared to the other sentiments (see Fig 4b). Notably, the structural breaks align closely with those of the full corpus, confirming the 2017–2018 shift reflects genuine changes in original discourse. Comparing the two conditions also shows that reposting disproportionately amplified neutral content (nearly 2 × the gain vs. originals), while negative posts showed the smallest gap between conditions, consistent with users preferentially resharing moderate over hostile messages.

### 3.2. Spatiotemporal sentiment analysis

We examine how sentiment varies across regions over time and identify regional differences in structural shifts. This analysis evaluates how the distribution of positive, neutral, and negative posts evolves across regions over time and identifies structural shifts in sentiment dynamics at the territorial level.

First, we analyzed the monthly number of posts of each sentiment across territorial units, focusing on the six territorial units with the highest concentrations of Venezuelan migrants as of December 2024 (see Table 5), and Fig 5 shows the monthly sentiment frequency for each territorial unit from 2014 to early 2020 together with the detected structural breaks.

**Table 5:** Distribution of Venezuelan Migrants by Territory (December 2024). Data source: Observatorio de Migraciones, Migrantes y Movilidad Humana (OM3) [33].

Territory	Number of Venezuelan Migrants	Share of National Venezuelan Migrant Population (%)
Bogotá D.C.	591,462	21.0%
Antioquia	390,936	13.9%
Norte de Santander	331,152	11.8%
Atlántico	199,436	7.1%
Valle del Cauca	198,311	7.0%
La Guajira	159,101	5.6%

**Fig 5:**
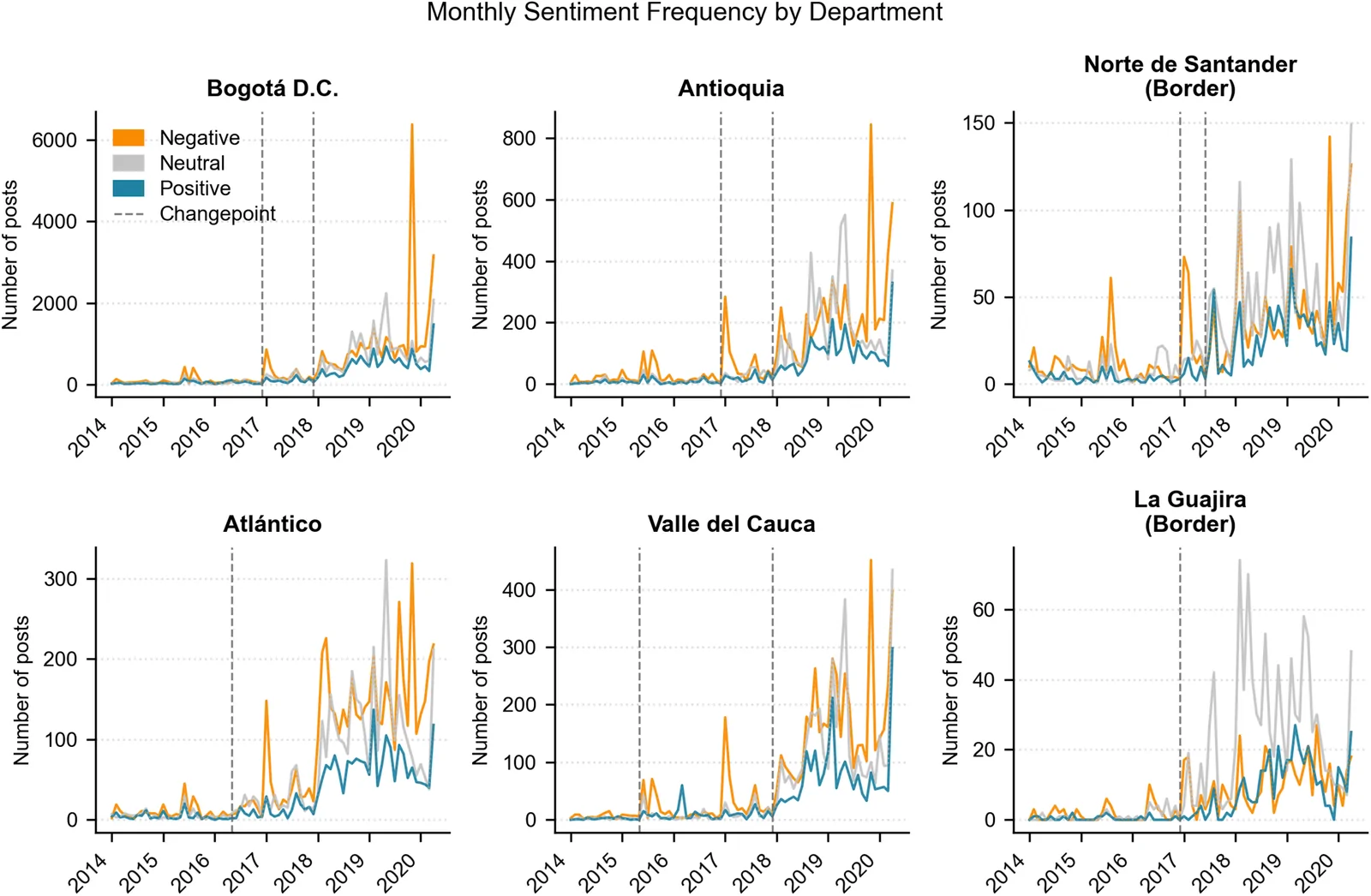
Monthly sentiment frequency by territorial unit with high concentrations of Venezuelan migrants (2014–2020). Vertical dashed lines indicate changepoints detected in the monthly series of negative posts.

Negative sentiment was dominant in five of the six territorial units analyzed, with La Guajira the sole exception; neutral posts consistently outnumbered negative ones there throughout the observation period, consistent with the more descriptive register of discourse in a border-adjacent department. Bogotá D.C. tracked national trends closely (see Fig 4a and Fig 4b above), but was not insulated from surges; it registered some of the largest single-month negative counts in the entire regional dataset. Norte de Santander showed the earliest regional sensitivity to the 2017 migration wave, while Atlántico and Valle del Cauca exhibited their most pronounced activity in 2018 and beyond.

To investigate regional heterogeneity in the temporal dynamics of sentiment, we applied the same changepoint detection procedure used in the national analysis [32], statistically identifying shifts in the mean level of monthly negative post counts. Table 6 reports the corresponding break dates that mark transitions between distinct regimes in the volume of sentiment expressed online.

**Table 6:** First changepoint-detected structural breaks in the monthly count of negative posts by Territorial Units.

Territorial Unit	Changepoint 1	p	Changepoint2	p
Bogotá D.C.	December 2016	<0.001	December 2017	<0.001
Antioquia	December 2016	<0.001	December 2017	<0.001
Norte de Santander (Border)	December 2016	<0.001	June 2017	<0.001
Atlántico	May 2016	<0.001	–	–
Valle del Cauca	May 2015	<0.001	December 2017	<0.001
La Guajira (Border)	December 2016	<0.001	–	–

Across the six territorial units, most exhibit significant structural breaks between 2016 and 2018, aligning with the broader national shift. However, the timing and number of changepoints vary across regions. Border territories such as Norte de Santander and La Guajira show earlier shifts beginning in late 2016, while Valle del Cauca exhibits the earliest break in May 2015. Other regions, including Bogotá D.C. and Antioquia, display multiple changepoints spanning 2016–2017, indicating a staggered but broadly synchronized transition in sentiment dynamics (Table 6).

Finally, beyond communication dynamics, structural regional conditions may also influence how migration is discussed online. To explore this possibility, we examined correlations between the proportion of posts of each sentiment category (normalized by department population size) and a set of demographic and socio-economic indicators. Fig 6 shows the population distribution across Colombian departments and the total number of posts by department, providing geographic context for the analysis.

**Fig 6:**
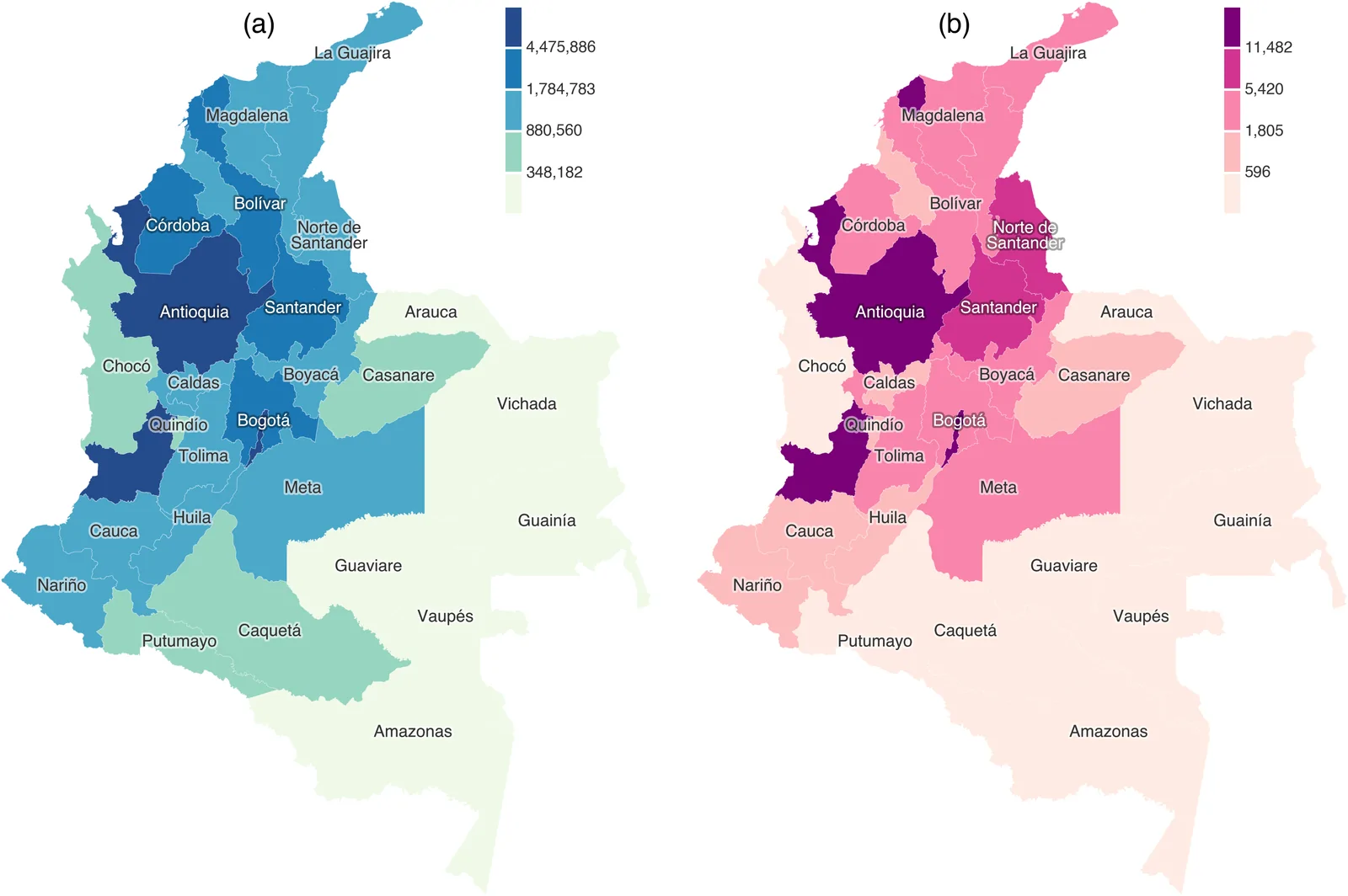
(a) Population of Colombia by departments in 2018 based on DANE [34], and (b) total number of posts per department. Department boundaries are based on the Marco Geoestadístico Nacional (MGN) published by DANE [35]. The figure was created by the authors using Python.

We conducted bivariate correlation analyses between sentiment proportions (normalized by department population) and a set of variables, including mugging incidents [36], unemployment [37], poverty rate [38], inequality [38], and homicide rates [39]. While most correlations were low in magnitude (r < 0.46), a few patterns are noteworthy (Table 7). Notably, the Gini coefficient and unemployment rate negatively correlated with the proportion of negative posts (r = −0.454 and r = −0.404, respectively), reaching statistical significance at the 5% level. These patterns indicate that departments with higher inequality and unemployment tend to exhibit a lower share of negative posts, although the underlying mechanisms remain unclear and provide opportunities for future investigation.

**Table 7:** Correlations between variables and the proportion of posts of each sentiment (by department).

Variable	Positive		Neutral		Negative	
	r	p	r	p	r	p
Number of mugging incidents per department in 2018	0.287	0.105	−0.368**	0.035	−0.031	0.864
Unemployment rate per department in 2018	0.261	0.218	−0.099	0.644	−0.404**	0.050
Poverty rate per department in 2018	−0.022	0.918	0.153	0.475	−0.310	0.140
Extreme poverty rate per department in 2018	−0.110	0.609	0.103	0.632	0.024	0.911
Gini coefficient per department in 2018	0.095	0.659	0.093	0.665	−0.454**	0.026
Number of homicides per department in 2018	0.261	0.149	−0.193	0.290	−0.133	0.467

^*^ p < 0.10, ^**^ p ≤ 0.05, ^***^ p ≤ 0.01.

*r* denotes the correlation coefficient and *p* the corresponding p-value.

### 3.3. Temporal analysis of pejorative terms

The temporal evolution of specific terms used to describe Venezuelan migrants in Colombia offers insight into how language adapts within public discourse and may contribute to processes of stigmatization. For this analysis, we analyzed the monthly and yearly distribution of posts containing the derogatory terms, as illustrated in Fig 7.

**Fig 7:**
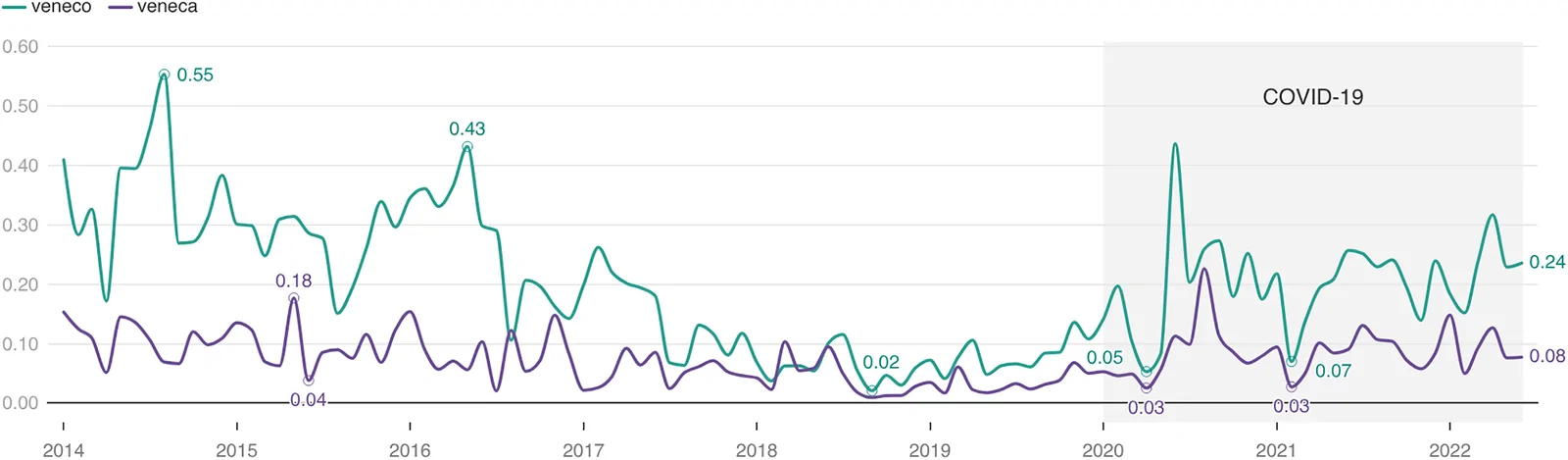
Monthly proportion of posts containing derogatory terms relative to the total number of migration related posts.

Across most periods, the masculine term appears more frequently than the feminine form, likely reflecting Spanish grammatical conventions in which masculine plurals can refer to mixed-gender groups, making it a more general reference. The use of both terms declines through mid-2018, stabilizes through 2019, and then increases again beginning in early 2020, with peaks during 2020–2021.

Fig 8 shows the proportions of posts containing these terms across four event-driven periods (pre-2017, 2017–2018, 2019, and COVID-2020). Despite regional variation in magnitude, the temporal pattern is consistent: usage increases substantially during the 2017–2018 period, the peak across most departments. These patterns sustain at high levels until the relative share of posts with these terms begin to decline during the COVID-19 period in 2020. This trajectory mirrors national trends, indicating a broad synchronization in how derogatory language emerges and recedes across regions in response to socio-political events.

**Fig 8:**
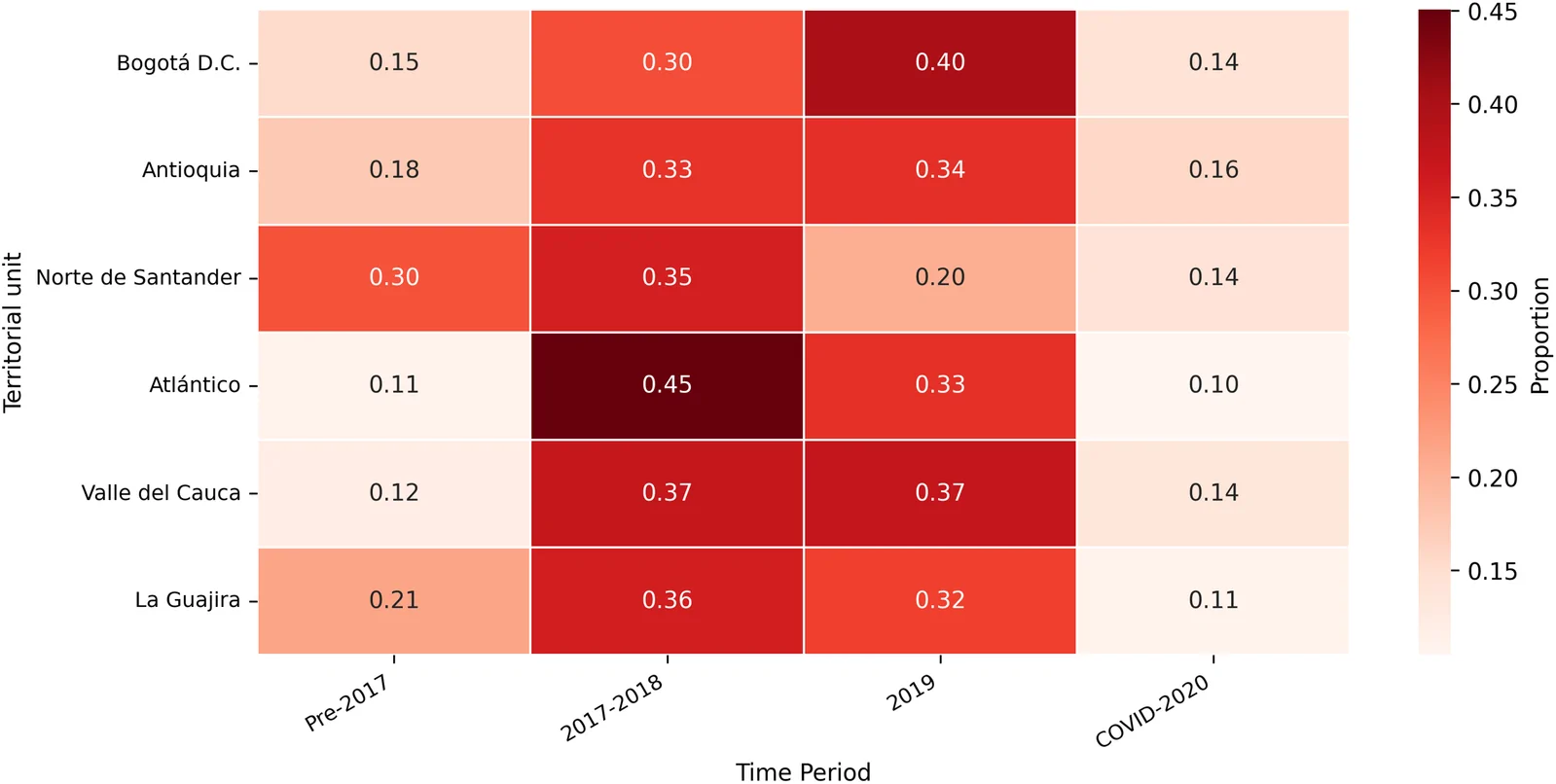
Regional distribution of derogatory term usage across Colombian territorial units by period.

We further examine how the sentiment associated with these terms evolves over time (monthly) by looking at the masculine and feminine terms that were classified as negative, relative to the total number of posts containing each term (Fig 9). Despite substantial month-to-month variability— particularly for the less frequently used *feminine term*—a broader temporal trend is visible. From 2015 to mid-2018, the average proportion of posts increased substantially for both terms (masculine from 54% to 82%; feminine from 72% to 95%), reaching peak levels during 2018−2019. Although negativity declines slightly after 2019 (to 73% masculine; 79% feminine), it remains elevated relative to pre-2018 levels, indicating a shift toward more negative usage over time.

**Fig 9:**
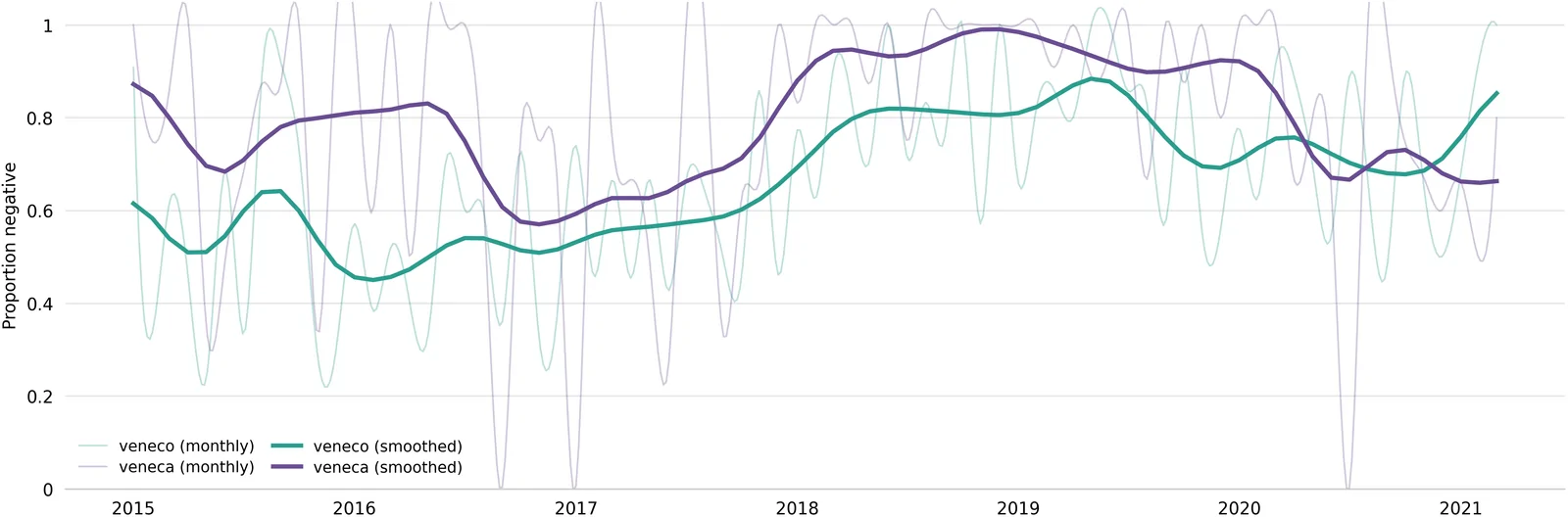
Monthly proportion of negative sentiment in posts containing the masculine and feminine derogatory terms. Thin lines show the monthly proportions, while thick lines represent a 3-month moving average.

### 3.4. Thematic evolution of migration discourse

To provide additional context, we applied topic modeling to identify broad thematic patterns in the discourse. Fig 10 presents the monthly prevalence of the eight topics identified by the model. Each line represents the proportion of posts associated with a given topic over time, while vertical markers indicate major sociopolitical events referenced in the analysis.

**Fig 10:**
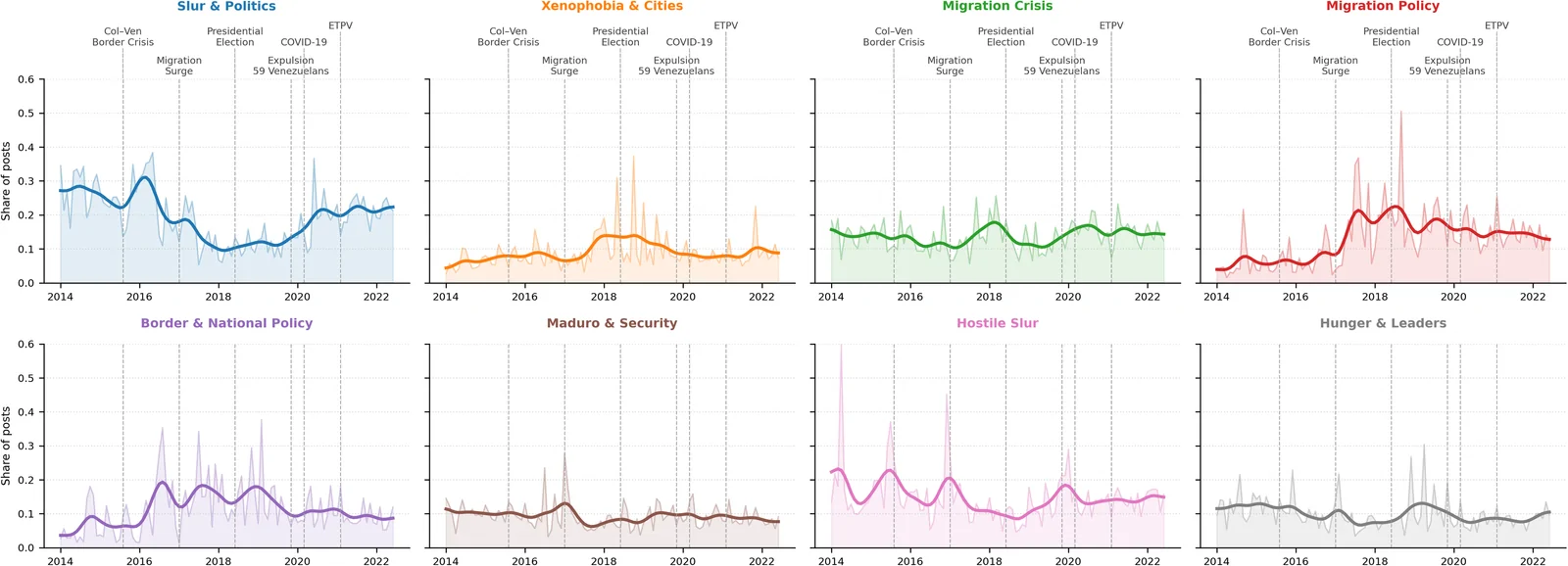
Monthly prevalence of LDA topics of posts from Colombia discussing Venezuelan migration.

The topics reflect several recurring narrative frames in the conversation about Venezuelan migration on X posts from Colombian, ranging from derogatory language directed at migrants (e.g., *Slur & Politics* and *Hostile Slur*) to policy and governance discussions (e.g., *Migration Policy* and *Border & National Policy*), as well as broader narratives about urban presence (e.g., *Xenophobia & Cities*, *Migration Crisis*, *Maduro & Security*, and *Hunger & Leaders*).

Topic prevalence varies over time. In the early years of the dataset, derogatory topics—particularly *Slur & Politics* and *Hostile Slur*—appear relatively prominent. Around 2017–2018, attention shifts towards topics related to migration scale and visibility, especially *Xenophobia & Cities* and *Migration Crisis*, alongside increased discussion of policy responses (e.g., *Migration Policy* and *Border & National Policy*). During the same period, some slur-related topics decline in relative prominence. This shift suggests a transition from discourse centered on derogatory labeling toward broader political and societal framing of migration, reflecting the incorporation of migration into national policy debates and public discourse. Coinciding with the structural changes identified in our temporal analysis, this shift points to a broader transformation in how migration is framed in public discourse.

It is important to note that topic assignment and sentiment classification are independent model outputs describing different properties of a post: topic modeling captures the lexical and thematic content of a post (e.g., the presence of slur-related or political vocabulary), while sentiment classification captures its affective polarity. A post belonging to a derogatory-keyword topic such as Slur & Politics or Hostile Slur can therefore still be classified as sentiment-neutral, for example when it discusses or quotes such language in a descriptive or factual register rather than expressing negative affect. This distinction reconciles the apparent tension between the prominence of derogatory topics in the early years of the dataset and the concurrent predominance of neutral sentiment reported in the temporal sentiment analysis (see “Temporal sentiment analysis” above): the two measures capture different dimensions of the same posts rather than conflicting estimates of the same construct.

## 4. Discussion

Four main findings emerge from the analysis, each highlighting a distinct aspect of how migration discourse is produced, circulated, and transformed on social media platforms.

First, platform amplification mechanisms (e.g., reposting) fundamentally reshape the sentiment of migration discourse on social media platforms. Original posts contain a substantially higher share of negative sentiment, while reposts disproportionately amplify neutral and positive content. This distinction indicates that expressed (original post) and amplified sentiment (reposts) are not equivalent: while users frequently produce negative content, the overall online community is more likely to circulate non-negative messages. As a result, the amplified sentiment of migration discourse visible on the platform may appear more moderate than the underlying distribution expressed by individual users. This pattern is consistent with prior research on information diffusion, which suggests that while emotionally charged messages may be widely produced, positive or neutral content can reach larger audiences amplification dynamics like reposts [40,41].

Second, the analysis identifies a structural shift in migration discourse beginning around 2017−2018. Changepoint detection reveals a transition from a lower-volume, more balanced discourse to a higher-volume regime in which negative sentiment becomes prominent and sustained until subsequent changepoints. Even when it decreases, it remains elevated from its original state. These shifts are observed across multiple sentiment categories and territorial units, indicating a broad transformation in how migration, particularly through the use of derogatory terms. The timing of these shifts coincide with socio-political events including the 2018 Colombian presidential election debates centered around migration [6], as well as the COVID-19 pandemic. In this sense, the results suggest that migration discourse does not simply react to events but can undergo more fundamental transitions in sentiment and scale as socio-political pressures accumulate, creating multiple changepoint shifts. This interpretation is consistent with prior work demonstrating Twitter/X as an informal barometer of public mood, sensitive to crises, political milestones, and policy shifts [42,43].

Third, the results highlight regional variation in the timing of migration discourse across Colombia. While national trends are broadly reflected across departments, structural shifts occur at different moments across regions, highlighting the importance of a spatio-temporal analysis. Border departments such as Norte de Santander and La Guajira are among the first regions to exhibit structural shifts in sentiment dynamics. These findings suggest that geographic context shapes how migration is experienced and discussed—particularly in relation to derogatory slurs—although evidence here is limited to temporal variation and does not establish a causal relationship. This finding nuances prior research finding little differences in sentiment across territorial units [44], with our research suggesting that such differences may emerge more clearly when examined over time rather than as a static comparison.

Fourth, the analysis reveals an evolution in the linguistic and thematic framing of migration discourse. The use of derogatory terms such as *veneco* and *veneca* fluctuates in response to broader social and political developments. While the overall frequency of these terms does not increase continuously, their association with negative sentiment becomes stronger over time, suggesting a semantic shift toward greater stigmatization [45,46]. This shift is notable given that these terms did not originally carry uniform pejorative connotations [47]. At the same time, topic modeling further showed that migration discourse evolves from early hostile rhetoric toward more politicized and policy-oriented debates, reflecting the incorporation of migration into broader national discussions about governance, security, and economic conditions. These findings are consistent with research on scapegoating and social stress, which link periods of political or economic uncertainty can intensify negative narratives toward migrant groups [48,49].

Although national patterns are broadly reflected across regions, the timing of these shifts varies, indicating spatial heterogeneity in how migration discourse evolves. Rather than moving linearly toward either hostility or solidarity, online discourse shifts in response to political events, regional contexts, and platform dynamics. In particular, reposting behavior, plays a critical role in shaping how online discourse is amplified and perceived, while structural shifts in sentiment and language over time indicate deeper transformation in how migration is collectively understood on social media platforms.

Two caveats apply to the interpretations offered above. First, sentiment as measured here reflects the emotional polarity of a post’s language and is not equivalent to an attitude toward migrants: negative sentiment may reflect hostility, but it may equally reflect concern for migrants’ welfare, criticism of government policy, or reporting on adverse events, and our sentiment classifier does not distinguish between these underlying motivations. Second, the structural shifts identified through changepoint detection describe statistically significant transitions in the volume and polarity of online discourse; they do not directly measure public opinion, social cohesion, or the absorptive capacity of host communities. Explanations invoking these broader constructs, including the discussion of absorptive capacity below, should therefore be read as possible interpretations consistent with the observed patterns rather than conclusions directly established by the data.

One way to interpret the structural shift identified in this analysis is through the lens of the absorptive capacity of host communities receiving sudden, large arrivals of migrants. The transition observed around 2017–2018—from a more balanced discourse to one in which negative sentiment becomes more prominent and sustained—may reflect a point at which social and institutional systems are less able to accommodate the pressures associated with large-scale migration. While this study does not directly measure absorptive capacity, the changepoint provides an observable signal of a broader shift in how migration is collectively perceived and discussed, consistent with the idea that public sentiment can change non-linearly as underlying pressures accumulate.

These findings also have several implications for policymakers and organizations working on migrant integration and social cohesion. First, monitoring large-scale social media discourse may help identify emerging shifts in online post sentiment toward migrants. In this study, spikes in negative sentiment frequently coincided with major political or social events, suggesting that online discourse can function as an informal signal of emerging social tensions. Second, the persistence and evolution of derogatory language highlight the importance of communication strategies that counter misinformation and stigmatizing narratives about migrants. Public messaging that emphasizes migrants’ contributions and shared social ties may help mitigate the spread of hostile narratives. Third, the regional variation observed in this study suggests that local context shapes migration discourse. Policy responses aimed at improving social cohesion may therefore benefit from regional tailoring rather than relying solely on national messaging strategies. Finally, the gendered patterns observed in online discourse highlight the importance of considering the gendered dimensions of migration narratives when designing communication strategies and integration initiatives.

## 5. Limitations

Although X provides large-scale, real-time data on online discourse, its user base is not demographically representative of the general population. The platform had approximately 4.3 million users in Colombia in early 2022, corresponding to about 8.4% of the national population [20], and X users tend to be younger and more educated than the broader public [21]. The patterns observed here therefore reflect the discourse of an active online community rather than the attitudes of the Colombian population as a whole.

A second limitation concerns geolocation data availability. Only a subset of posts contained geographic coordinates, restricting the spatial coverage of the spatiotemporal analysis. Regional patterns should be interpreted as indicative rather than comprehensive.

A related limitation concerns the geolocation classifier used to infer location for posts without user-provided location data. Although this classifier’s precision, recall, and F1-score (~0.79–0.80; see Methodology) are reasonable, applying it to millions of posts implies a non-negligible number of misclassifications, and because the classifier is trained on post text, it is possible that it partly captures topic-specific or politically inflected vocabulary correlated with, rather than diagnostic of, a post’s true geographic origin. This circularity concern does not apply to the study’s central national-level temporal findings or to the department-level analysis: the robustness check reported above (Results; S1 Fig) reproduces identical structural breaks using only directly-geolocated posts, whose location was never derived from post text, and the department-level analysis (Fig 5, Table 6) was likewise built entirely from directly-geolocated posts. Both checks are nonetheless bounded to the 2014−01–2020−04 window covered by directly-geolocated data, and do not address possible circularity in the remaining geolocation-dependent analyses in this paper (the overall sentiment trends beyond the changepoints, the topic model, and the derogatory-term analysis), which continue to rely on the full corpus, including inferred locations, nor can they speak to the 2020–2022 portion of the study period.

Finally, computational methods such as sentiment classification and topic modeling may not fully capture contextual nuances like irony or sarcasm. Results should be read as aggregated patterns in online discourse rather than precise measurements of individual attitudes.

This study’s analytical pipeline also relies on a sequence of independently trained classifiers (geolocation, relevance, sentiment) and a topic model, each of which introduces its own classification error; the output of one stage is used as input to the next, and our analyses treat these labels as given rather than formally modeling how error at each stage propagates through the pipeline. Relatedly, we did not assess whether classifier performance is stable across the 2014–2022 study period, as the annotated evaluation samples (a few hundred to roughly 1,000 posts per task) are too small to support a reliable year-by-year breakdown. We also did not attempt to detect bots or coordinated inauthentic activity, which could disproportionately affect sentiment or topic prevalence at particular points in time. Addressing error propagation across the pipeline, temporal stability of model performance, and the potential influence of bots or coordinated activity are important directions for future work.

## 6. Conclusions

This study provides a multidimensional analysis of migration discourse for the case of Venezuelan migrants in Colombia, which evolves over time, across regions, and in response to socio-political events. The findings show that original posts tend to be more negative, while reposting amplifies more neutral or positive content, indicating that what circulates on social media most widely does not directly mirror the sentiment of many original content creators. This analysis also identifies multiple statistically significant shifts in the sentiment of discourse that correspond with major socio-political events. A broader transition began for most districts around 2017–2018, shifting toward more consistently negative sentiment across regions. In addition, the sentiment associated with commonly used derogatory slur terms becomes more negative over time. While all districts appear to experience similar shifts, we found that the timing of these shifts varies between districts, which is an area for future investigation. Together, these results show that migration discourse is shaped not only by what people say, but also how content is amplified, and that it evolves through both event-driven shifts and longer-term lasting transformations in how migration is framed online.

## Supporting information

S1 Text NLP models used for geolocation, relevance filtering, and sentiment classification. (DOCX)

S2 Text Latent Dirichlet Allocation topic model, corpus, parameters, and results. (DOCX)

S1 Fig Monthly sentiment counts for directly-geolocated posts only (2014−01–2020−04), with detected changepoints, compared to the full-corpus results in Table 4. (PNG)
