# Supplementary material for: Multidimensional analysis of social discourse on Twitter (X) around Venezuelan migration in Colombia
Source: PLoS One. 2026 Oct 14;21(10):e0360050. doi: 10.1371/journal.pone.0360050 (PMC13667793; doi:10.1371/journal.pone.0360050)
Supplement: Supplementary file 1 [file pone.0360050.s001.docx]

### S1 Text. NLP models used for geolocation, relevance filtering, and sentiment classification

This Supporting Information provides detailed descriptions of the natural language processing (NLP) models used throughout the study, including their architecture, pre-training data, fine-tuning procedures, key parameters, and classification examples. These details complement the methodological descriptions provided in Sections 3.2 and 3.3 of the main manuscript.

### S1A. Overview of NLP tasks and models

Three NLP tasks were central to this study: (i) geolocation inference, (ii) relevance validation, and (iii) sentiment classification. For each task, multiple models were evaluated to ensure robustness and to identify the best-performing architecture. Across all tasks, datasets were split into training and testing subsets using a 70/30 ratio, a commonly adopted practice in neural network–based classification that balances model learning and generalization performance [1]. Model performance was primarily assessed using classification accuracy on held-out test sets, as reported in Tables 1–3 of the main manuscript.

**Classification examples.**

| **Task** | **Spanish post (truncated)** | **Gold label** | **Chosen model prediction** |
| --- | --- | --- | --- |
| Geolocation | “De Iquitos sus venezolanos. Los migrantes llaneros llegaron a la selva peruana buscando un nuevo futuro.” | Other country | Other country |
|  | “En Colombia, hasta septiembre de 2018, había 1.032.016 venezolanos de acuerdo con Migración Colombia” | Other country | Colombia |
|  | “IvanDuque Que pasa con la frontera con Venezuela, donde esta el supuesto control y la supuesta restricción Supuestamente no están cerradas las fronteras” | Colombia | Colombia |
| Relevance | “La fila mas absurda de la vida (Migración Colombia en Bogotá)” | Irrelevant | Irrelevant |
|  | “Respetemos a los migrantes venezolanos o de cualquier nacionalidad que se empleen como repartidores 🚴 y apoyemos con propinas 💰 su valioso trabajo.” | Relevant | Relevant |
|  | “Mujeres migrantes huyeron del hambre en Venezuela y se prostituyen en Colombia para alimentar a sus familias - Reportaje📝” | Relevant | Relevant |
| Sentiment | “veneco que veo, veneco que pateo.” | Negative | Negative |
|  | “Un informe de Migración señala que más de 870 mil ciudadanos venezolanos están radicados en Colombia. Vea en dónde están concentrados.” | Neutral | Neutral |
|  | “Un arduo trabajo con nuestros hermanos migrantes y refugiados. Con una atención integral: 1. Formación en valores ciudadanos y democracia, prevención de enfermedades de transmisión sexual, derechos y deberes 2. Atención al migrante 3. Atención en salud” | Positive | Positive |

### S1B. spaCy es_core_news_sm

**Model description.**

The es_core_news_sm model is a lightweight Spanish-language NLP pipeline provided by spaCy, designed for efficient processing of short texts such as social media posts [2]. It includes components for tokenization, morphological analysis, lemmatization, dependency parsing, and named-entity recognition, as well as vector representations suitable for downstream classification tasks.

Es_core_news_sm is not transformer-based, instead it relies on a shallow neural network optimized for speed and stability rather than deep contextual representation. The model is pretrained on a mixture of Spanish web and news text curated by spaCy and is intended to provide broad coverage of contemporary Spanish usage.

**Fine-tuning and performance.**

This model was fine-tuned for two tasks in this study:

- **Geolocation** (Section 3.2.1): binary classification of posts as originating from Colombia or another country, using a training set of 270,000 posts labeled via the Nominatim API [3].
- **Relevance** (Section 3.2.2): binary classification of posts as relevant or irrelevant to Venezuelan migration, using a balanced dataset of 3,108 manually labeled posts.
- **Sentiment classification** (Section 3.3.1): multi-class classification of post sentiment (negative, neutral, positive), trained on a manually annotated subset of 1,920 posts.

Default spaCy optimization settings were used, with cross-entropy loss and minibatch stochastic gradient descent. The model achieved an accuracy of 0.79 for geolocation, and 0.90 for relevance validation, outperforming alternative transformer-based models in both tasks (Tables 1 and 2). However, it only achieved an accuracy of 0.552 for sentiment classification, the lowest of the three models.

### S1C. BETO

**Model description.**

BETO is a transformer-based language model derived from the BERT architecture and pretrained exclusively on large-scale Spanish corpora [4]. It follows a BERT-base configuration with 12 transformer layers and uses a whole-word masking strategy during pre-training, which improves semantic representation in morphologically rich languages such as Spanish.

The model was pretrained on a combination of Spanish Wikipedia, news articles, and other large text collections, enabling it to capture nuanced syntactic and semantic relationships in Spanish discourse. Owing to its language-specific pre-training, BETO is particularly well suited for tasks requiring fine-grained interpretation of sentiment.

**Fine-tuning and performance.**

BETO was fine-tuned for two classification tasks in this study:

- **Geolocation** (Section 3.2.1): binary classification of posts as originating from Colombia or another country, using a training set of 270,000 posts.
- **Relevance validation (Section 3.2.2):** binary classification of posts as relevant or irrelevant to Venezuelan migration, using a balanced dataset of 3,108 manually labeled posts.
- **Sentiment classification (Section 3.3.1):** multi-class classification of post sentiment (negative, neutral, positive), trained on a manually annotated subset of 1,920 posts.

Training followed a 70/30 train–test split with cross-entropy loss and standard transformer optimization procedures. BETO achieved an accuracy of 0.5 in Geolocation (Table 1), 0.81 in relevance validation (Table 2) and the highest accuracy among evaluated models for sentiment classification (0.699; Table 3). Due to its superior performance on sentiment tasks, BETO was selected to label the full corpus of migration-related posts by sentiment.

### S1D. Multilingual BERT (bert-base-multilingual-uncased-sentiment)

**Model description.**

The bert-base-multilingual-uncased-sentiment model is based on the multilingual BERT architecture, which was pretrained on text from over 100 languages using a shared subword vocabulary [5]. It adopts the standard BERT-base encoder structure and is fine-tuned for sentiment classification through the addition of a task-specific output layer.

Because of its multilingual pre-training, this model is capable of cross-lingual generalization and is commonly used in settings where language usage may vary or include mixed linguistic signals.

**Fine-tuning and performance.**

This model was fine-tuned for two classification tasks in this study:

- **Relevance validation (Section 3.2.2):** binary classification of posts as relevant or irrelevant to Venezuelan migration, using a balanced dataset of 3,108 manually labeled posts.
- **Sentiment classification (Section 3.3.1):** multi-class classification of post sentiment (negative, neutral, positive), trained on a manually annotated subset of 1,920 posts.

We used the same balanced dataset of 3,108 manually labeled posts and the same 70/30 train–test split applied to the other models. Training employed cross-entropy loss and standard optimization procedures for transformer models.

The multilingual BERT model achieved an accuracy of 0.84 in relevance classification (Table 2), and 0.666 for sentiment classification, underperforming relative to spaCy’s *es_core_news_sm* and BETO respectively. As a result, it was retained as a comparative benchmark but not selected for the automatic labeling.

References

1.	Nguyen QH, Ly H-B, Ho LS, Al-Ansari N, Le HV, Tran VQ, et al. Influence of Data Splitting on Performance of Machine Learning Models in Prediction of Shear Strength of Soil. Mathematical Problems in Engineering. 2021;2021:4832864. doi: 10.1155/2021/4832864.

2.	es_core_news_sm SpaCy: Explosion AI; 2023. Available from: <https://spacy.io/models/es#es_core_news_sm>.

3.	Nominatim GitHub2023. Available from: <https://github.com/osm-search/Nominatim>.

4.	Cañete J, Chaperon G, Fuentes R, Ho J-H, Kang H, Pérez J. Spanish Pre-Trained BERT Model and Evaluation Data. 2020.

5.	Town N. bert-base-multilingual-uncased-sentiment (Revision edd66ab): Hugging Face; 2023. Available from: <https://huggingface.co/nlptown/bert-base-multilingual-uncased-sentiment>.
