# Supplementary material for: Multidimensional analysis of social discourse on Twitter (X) around Venezuelan migration in Colombia
Source: PLoS One. 2026 Oct 14;21(10):e0360050. doi: 10.1371/journal.pone.0360050 (PMC13667793; doi:10.1371/journal.pone.0360050)
Supplement: Supplementary file 2 [file pone.0360050.s002.docx]

### S2 Text. Latent Dirichlet Allocation topic model: corpus, parameters, and results

This Supporting Information provides a full description of the Latent Dirichlet Allocation (LDA) [1] topic model applied to the corpus of Colombian Twitter posts about Venezuelan migration. It covers the data used for model training, text preprocessing steps, vectorization and model parameters, coherence-based topic selection, topic descriptions, temporal dynamics, and statistical tests for period-level differences. These details complement the methodological descriptions in Section 3.4 of the main manuscript.

### S2A. Corpus and sampling

The topic model was fitted on original posts only, retweets (identified by the prefix 'RT @') were excluded to avoid overrepresentation of individual messages. The starting corpus comprised 1,664,903 posts in the full validated dataset.

Because LDA training on the full corpus is computationally prohibitive, a stratified random sample of 200,000 original posts was drawn prior to fitting. Sampling was proportional to each calendar month's share of the total original-post volume, ensuring that the distribution of posts across time was preserved in the training set. The NumPy random generator was seeded at 42 to guarantee reproducibility.

After fitting on the sample, the trained topic model was applied to the entire original-posts corpus to obtain topic assignments for all posts, enabling the construction of a monthly topic-proportion matrix spanning January 2014 to December 2022.

### S2B. Text preprocessing

Each post was preprocessed through the following sequential steps:

- Lowercasing: all characters were converted to lowercase.
- URL removal: HTTP and HTTPS URLs were replaced with whitespace using the pattern http\S+|www\S+.
- Mention removal: Twitter @-handles were replaced with whitespace using the pattern @\w+.
- Non-alphabetic removal: all characters other than unaccented and accented Spanish letters (a–z, á, é, í, ó, ú, ü, ñ) were removed, stripping punctuation, digits, and special characters.
- Whitespace normalisation: multiple consecutive whitespace characters were collapsed to a single space.

No stemming or lemmatisation was applied; words were retained in their surface form to preserve the distinction between singular and plural slur terms (veneco vs. venecos, veneca vs. venecas), which carry different thematic connotations.

### S2C. Stopword removal and vocabulary construction

Stopwords were drawn from two sources: (i) the NLTK Spanish stopword list, and (ii) the NLTK English stopword list, to cover any English-language posts in the corpus. These were supplemented with a custom list of high-frequency Twitter-specific tokens and demonym variants that carry no discriminative thematic information:

- Twitter noise: rt, via, amp
- High-frequency Spanish function words not covered by NLTK: que, los, las, del, por, con, una, uno, para, como, más, pero, este, esta, son, está, han, ser, fue, si, ya, cuando, hay, solo, puede, bien, porque, qué, así, también, ese, eso, esa, va, yo, mi, sus
- Demonyms and country names: colombia, venezuela, venezolanos, venezolano, venezolana, venezolanas, colombiano, colombianos, colombiana, colombianas (excluded because they appear in virtually every post and carry no discriminative topic signal)

After stopword removal, the vocabulary was constructed using scikit-learn's CountVectorizer [2] with the following parameters:

- token_pattern: [a-záéíóúüñ]{3,} — tokens must be at least three characters and contain only Spanish-valid alphabetic characters
- max_features: 8,000 — the vocabulary is capped at the 8,000 most frequent terms after stopword filtering
- min_df: 5 — terms appearing in fewer than five documents in the training sample are excluded

These settings resulted in a document-term matrix of 200,000 posts (rows) × 8,000 vocabulary terms (columns) used for model fitting.

### S2D. LDA model parameters

Topic modelling was performed using scikit-learn's LatentDirichletAllocation class. The following parameters were fixed across all candidate models:

- learning_method: 'online' — online variational Bayes inference, suitable for large corpora
- max_iter: 20 — maximum number of EM iterations
- random_state: 42 — fixed seed for reproducibility
- All other hyperparameters (including the Dirichlet priors α and β) were left at scikit-learn defaults

Candidate models were fitted for N ∈ {6, 7, 8, 9, 10} topics. For each N, a separate LDA model was trained on the same document-term matrix.

### S2E. Coherence-based topic selection

Model quality was assessed using the cᵥ coherence metric [3], computed via Gensim's CoherenceModel. The cᵥ measure evaluates the semantic coherence of each topic's top-10 keywords by combining normalised pointwise mutual information (NPMI) and a sliding-window context model, averaging over all topic-word pairs. Higher values indicate more interpretable, coherent topics.

For coherence computation, the document-term vocabulary was additionally filtered using Gensim's Dictionary.filter_extremes with no_below = 5 and no_above = 0.95, to remove very rare and very common terms from the coherence calculation. The coherence scores for each candidate model are reported in Table S2-1.

Table S2-1. Coherence scores (cᵥ) for candidate LDA models.

| **Number of topics (N)** | **Coherence score (cᵥ)** |
| --- | --- |
| 6 | 0.284 |
| 7 | 0.284 |
| 8 | **0.327** |
| 9 | 0.293 |
| 10 | 0.295 |

The 8-topic model achieved the highest coherence score (cᵥ = 0.327) and was selected for all subsequent analyses.

### S2F. Topic descriptions and keyword profiles

The eight topics identified by the selected model are summarised in Table S2-2, together with their assigned descriptive labels and top-10 keywords. Labels were assigned by the research team after inspection of the keyword profiles and a random sample of representative posts for each topic.

Table S2-2. Top 10 keywords and descriptive labels for the eight LDA topics.

| **Topic** | **Label** | **Top keywords (Spanish)** |
| --- | --- | --- |
| 1 | Slur & Politics | veneco, duque, claudialopez, mil, mundo, internacional, gracias, refugiados, pueblo, vida |
| 2 | Xenophobia & Cities | bogotá, ivanduque, personas, xenofobia, cómo, cúcuta, dictadura, niños, toda, ciudad |
| 3 | Veneca & Migration | veneca, millones, venecas, migración, crisis, madre, debe, cuenta, bueno, decir |
| 4 | Migration Policy | migrantes, hoy, migrante, presidente, migración, quiero, población, gobierno, día, protección |
| 5 | Border & National Policy | migración, país, frontera, atención, dice, gobierno, nacional, año, ciudadanos, según |
| 6 | Maduro & Security | aquí, menos, mejor, claudia, guardias, siempre, mientras, lópez, video, maduro |
| 7 | Hostile Slur | venecos, gente, ahora, situación, hace, ver, mierda, van, nunca, cada |
| 8 | Hunger & Leaders | venecos, maduro, petro, parte, hambre, gran, dijo, acá, ahora, mas |

Detailed descriptions of each topic are provided below.

#### Topic 1: Slur & Politics

Posts in this topic combine the masculine slur veneco with references to national political figures (President Duque, Bogotá mayor Claudia López) and humanitarian framing (refugiados, internacional). The topic reflects the politicization of Venezuelan migration in mainstream public discourse, where hostile terminology co-occurs with policy and civic vocabulary. The high probability loading on veneco (0.191) confirms this topic as the primary locus of slur usage in politically framed posts.

#### Topic 2: Xenophobia & Cities

This topic explicitly names xenophobia and links it geographically to the two Colombian cities most exposed to migration (Bogotá and Cúcuta). References to dictadura and niños embed the discourse in a political and humanitarian framing, while ivanduque (president Ivan Duque) indicates that this topic is partly organized around presidential-level political debate. The topic is more episodic than Topic 1, concentrating around specific political events.

#### Topic 3: Veneca & Migration

Dominated by the female form of the slur (veneca, venecas), this topic is distinct in its gendered targeting of Venezuelan women. The co-occurrence of millones and crisis situates the slur within narratives of migration scale, while madre introduces a familial framing that may reflect both humanitarian and derogatory registers. This topic is the primary site of gender-specific hostile discourse in the corpus.

#### Topic 4: Migration Policy

This topic centres on formal policy language: migrants as a population category (migrantes, migrante), government responses (gobierno, presidente), and protection frameworks (protección, población). It reflects institutional discourse rather than emotional or hostile framing and shows a structural increase following 2017 consistent with the formalisation of Colombian migration governance.

#### Topic 5: Border & National Policy

Focused on the national-level border question: migration flows (migración), the frontier (frontera), and national belonging (ciudadanos, nacional). This topic captures ongoing debate around border management and national identity, and shows slow but sustained growth throughout the observation window, reflecting the progressive institutionalisation of border-framing as the migration crisis became structurally entrenched.

#### Topic 6: Maduro & Security

Claudia López (Bogotá's mayor) appears alongside guardias (security forces) and video, suggesting this topic captures local security incidents involving Venezuelan migrants in Bogotá, often circulated via video and linked to municipal policy responses. The presence of maduro as a keyword indicates that these urban security incidents are contextualised within broader commentary on Venezuelan political leadership.

#### Topic 7: Hostile Slur

The most explicitly hostile topic in the model: venecos (plural masculine slur) paired with vulgar language (mierda) and vague situational framing (gente, situación, hace). Posts in this topic represent raw xenophobic expression with minimal political or policy content. The topic shows pronounced event-driven spikes around key crisis moments, consistent with scapegoating dynamics.

#### Topic 8: Hunger & Leaders

Links the masculine plural slur to political figures on both sides of the migration corridor, Maduro (Venezuela) and Petro (Colombia), alongside hambre (hunger) and reported speech markers (dijo). This topic reflects discourse connecting Venezuelan migration to political failure and food insecurity, often through reported statements rather than original commentary.

### S2G. Temporal dynamics

After fitting the model on the training sample, topic probabilities were computed for all original posts in the full corpus using the fitted vectorizer and LDA model. Topic assignments were aggregated by calendar month to produce a topic-month matrix of proportions (each row summing to 1.0), covering January 2014 to December 2022.

Temporal dynamics were visualized as a small-multiples (faceted) figure with eight panels, one per topic, showing monthly topic proportions as a raw time series with a Gaussian-smoothed trend overlaid (σ = 2 months). Six key socio-political events are marked with vertical dashed lines:

- August 2015: Colombia–Venezuela border crisis (mass expulsion of Colombians from Venezuela)
- January 2017: onset of mass Venezuelan emigration to Colombia
- June 2018: Colombian presidential election
- November 2019: expulsion of 59 Venezuelan nationals from Colombia
- March 2020: onset of COVID-19 pandemic
- February 2021: Colombia's Temporary Protection Statute (ETPV) for Venezuelan migrants

The smoothed trajectories revealed three qualitatively distinct temporal archetypes. Topics 1 (Slur & Politics) and 7 (Hostile Slur) are event-responsive: they amplify sharply at each marked crisis moment and recede between events, consistent with scapegoating dynamics. Topics 4 (Migration Policy) and 5 (Border & National Policy) are structurally increasing: they undergo a permanent level shift following 2016–2017 and do not return to their pre-migration-crisis baseline, reflecting the institutionalization of migration as a sustained political concern. Topics 3 (Veneca & Migration) and 6 (Maduro & Security) are static and chronic: they maintain a low, stable share regardless of external events, suggesting they function as persistent background informational registers. Topics 2 (Xenophobia & Cities) and 8 (Hunger & Leaders) occupy intermediate positions, showing moderate event-sensitivity without the sharp spikes of the hostile register or the structural permanence of the policy register.

### S2H. Statistical tests for period-level differences

To test whether topic proportions differed significantly across key periods of the migration crisis, a Kruskal-Wallis H test [4] was applied to the three most prevalent topics in the corpus (Topics 1, 7, and 3, identified by their mean monthly share). The five event periods used as groups were: Pre-2017 (January 2014–December 2016), 2017–2018 (January 2017–December 2018), 2019 (January–December 2019), COVID-2020 (January–December 2020), and ETPV-2021 (January–December 2021). Each group consisted of the monthly topic-proportion observations within that period. The Kruskal-Wallis test was selected because topic proportions are non-normally distributed and the group sizes are unequal.

Table S2-3. Kruskal-Wallis H test results for the three most prevalent topics across five event periods.

| **Topic** | **Label** | **Kruskal-Wallis H** | **p-value** |
| --- | --- | --- | --- |
| Topic 1 | Slur & Politics | 47.36 | < 0.001 |
| Topic 7 | Hostile Slur | 12.02 | 0.017 |
| Topic 3 | Veneca & Migration | 10.37 | 0.035 |

All three topics showed statistically significant variation across periods (p < 0.05), confirming that the temporal patterns observed in the smoothed figure reflect genuine distributional shifts rather than noise. The largest effect was observed for Topic 1 (Slur & Politics; H = 47.36, p < 0.001), indicating that politically framed slur usage was highly sensitive to the socio-political event structure of the migration crisis.

### References

1.	Blei DM, Ng AY, Jordan MI. Latent dirichlet allocation. Journal of machine Learning research. 2003;3(Jan):993-1022.

2.	Pedregosa F, Varoquaux G, Gramfort A, Michel V, Thirion B, Grisel O, et al. Scikit-learn: Machine learning in Python. the Journal of machine Learning research. 2011;12:2825-30. doi: 10.48550/arXiv.1201.0490.

3.	Röder M, Both A, Hinneburg A, editors. Exploring the space of topic coherence measures. Proceedings of the eighth ACM international conference on Web search and data mining; 2015.

4.	Kruskal WH, Wallis WA. Use of ranks in one-criterion variance analysis. Journal of the American statistical Association. 1952;47(260):583-621. doi: 10.1080/01621459.1952.10483441.
